# Splenic glucocorticoid resistance following psychosocial stress requires physical injury

**DOI:** 10.1038/s41598-017-15897-2

**Published:** 2017-11-16

**Authors:** Sandra Foertsch, Andrea M. Füchsl, Sandra D. Faller, Hannah Hölzer, Dominik Langgartner, Joanna Messmann, Gudrun Strauß, Stefan O. Reber

**Affiliations:** 1 Laboratory for Molecular Psychosomatics, Clinic for Psychosomatic Medicine and Psychotherapy, University Ulm, Ulm, Germany; 2 Institute of Physiology, University of Regensburg, Regensburg, Germany; 3 Department of Pediatrics and Adolescent Medicine, University Medical Center Ulm, Ulm, Germany

## Abstract

Mice exposed to chronic subordinate colony housing (CSC) stress show glucocorticoid (GC) resistance of *in vitro* lipopolysaccharide (LPS)-stimulated splenocytes, increased anxiety and colitis. Similar effects were reported in wounded mice exposed to social disruption (SDR). Here we show that CSC exposure induced GC resistance in isolated and *in* vitro LPS-stimulated, but not unstimulated, splenocytes, and these effects were absent when CD11b^+^ splenocytes were depleted. Moreover, re-active coping behaviour during CSC correlated with the attacks and bites received by the resident, which in turn highly correlated with the dimension of splenic GC resistance, as with basal and LPS-induced *in vitro* splenocyte viability. Importantly, social stress promoted spleen cell activation, independent of bite wounds or CD11b^+^/CD11b^−^ cell phenotype, whereas GC resistance was dependent on both bite wounds and the presence of CD11b^+^ cells. Together, our findings indicate that the mechanisms underlying splenic immune activation and GC resistance following social stress in male mice are paradigm independent and, to a large extent, dependent on wounding, which, in turn, is associated with a re-active coping style.

## Introduction

Chronically-stressed individuals run the risk for developing numerous somatic and affective disorders^1,2^, of which most are associated with an activated immune status and chronic low-grade inflammation^3–5^. Importantly, results from both pre-clinical and clinical studies are consistent with the hypothesis that an overshooting immune response to an acute stressor or trauma is even causally involved in the development of stress-related mood and somatic pathologies^6–9^. In line with these studies, we recently showed in mice that promoting regulatory T cell (Treg)-dependent immunoregulation prior to chronic psychosocial stress has stress-protective effects, preventing stress-induced anxiety and colitis^10,11^.

Given the well-known immunosuppressive effects of high doses of glucocorticoids (GC)^12^, the main effector molecules of the hypothalamus-pituitary-adrenal (HPA) axis, one possible mechanism underlying stress-induced chronic low-grade inflammation is development of GC resistance in certain immune cell subpopulations^13,14^. In support, caregivers for a family member with cancer showed a profound linear increase in systemic inflammation over time, as indexed by serum C-reactive protein (CRP), paralleled by *in vitro* GC insensitivity measured in whole blood incubated with lipopolysaccharide (LPS) and different concentrations of hydrocortisone^5^. Moreover, white blood cells from physically and emotionally healthy, but chronically-stressed, school teachers showed higher LPS-stimulated production and lower GC sensitivity of interleukin-6 (IL-6) secretion *in vitro* ^15^.

Splenomegaly and splenic GC resistance in *in vitro* Concanavalin A (Con A) - or LPS-stimulated spleen cells has also been reported following exposure to social disruption (SDR)^16–18^. During SDR an aggressive intruder mouse is introduced into the home cage of groups of 3 to 6 mice for 1 to 2 hours during their active phase on six consecutive days, while control groups remain undisturbed in their home cages^19^. In terms of the underlying mechanism these animal studies revealed a central role of GC resistant CD11b^+^ cells migrating from the bone marrow to the spleen^20,21^. Moreover, they showed that GC resistance in these cells is likely mediated by a combination of decreased GC receptor (GR) expression and a compromised GR nuclear translocation^22,23^. Of particular importance for the current study is the finding that GC resistance in *in vitro* LPS-stimulated splenocytes was only detectable in wounded, but not in non-wounded, SDR mice^19^, suggesting that the bite wounds received during social encounters play a central role in the development of SDR-associated GC resistance. Thus, although a lack of GC resistance following immobilisation stress^18^ initially suggested this phenomenon to be specific for social stressors, it rather seems as if the occurrence of skin injuries paralleling a certain social stressor, which in contrast to humans poses a common phenomenon in animals, and not the social character of the stressor per se, is critical for splenic immune activation and GC resistance. Support for this hypothesis is provided by the finding that splenocytes of wounded SDR mice produced more cytokines in response to LPS stimulation and showed a lower sensitivity to corticosterone (CORT) compared with mice exposed to a milder social stress paradigm preventing direct social interaction of the conspecifics and, thus, the occurrence of bite wounds^24^. However, it remains to be shown whether the mechanisms underlying SDR-induced splenic GC resistance are paradigm specific or of general relevance for social stressors allowing direct physical interaction. It is further not clear to date whether it is more a kind of “on-off” phenomenon or whether the dimension of spleen cell activation and GC resistance is dependent on the severity of stress-associated wounding. Moreover, although a positive correlation between submissive upright behavior during SDR exposure and the dimension of GC resistance suggests this kind of stress coping behavior to favour getting bitten by the dominant animal^19,25^, more detailed analyses of the behavioral stress coping strategies and severity of bite wounds, at best employing a social stress paradigm different from the SDR model, are required to understand these complex interactions and to generalize the mechanisms and effects. As in humans social stressors usually are not accompanied by skin injuries or wounding – despite certain occupational groups, as for instance policemen or soldiers on active duty, clearly have an increased risk to face injury in the context of social stress – it remains unclear whether there is bite wound independent spleen cells activation and GC resistance during social stress, contributing to systemic low-grade inflammation described in animals and humans following chronic stress.

In the present study we, therefore, exploit the chronic subordinate colony housing (CSC) paradigm, which poses another pre-clinically validated mouse model of chronic psychosocial stress^3^, to address these open questions. During CSC, four experimental mice are housed in direct physical contact with a larger, dominant male mouse for 19 consecutive days, leading amongst others to increased anxiety-related behavior and colitis^26–28^. Of special note is the fact that, although mechanistically not investigated so far, CSC mice also show a reduced GC sensitivity in isolated and *in vitro* LPS-stimulated splenocytes^3,27^. Furthermore, as published earlier it is possible to analyse and quantify in detail the individual behavioral coping strategies of each CSC mouse, distinguishing between pro-active stress coping, characterized by behaviors like attacking, chasing and mounting, and re-active stress coping, characterized by behaviours like flight, avoiding, submissive upright and scouting^10,29^. Intriguingly, shifting the coping style towards a more pro-active one has recently been shown to be associated with a lack of CSC-induced colitis and anxiety^10^, suggesting this kind of stress coping strategy to promote stress resilience.

## Results

### Individual behavioral stress coping during CSC exposure

**Figure 1:**
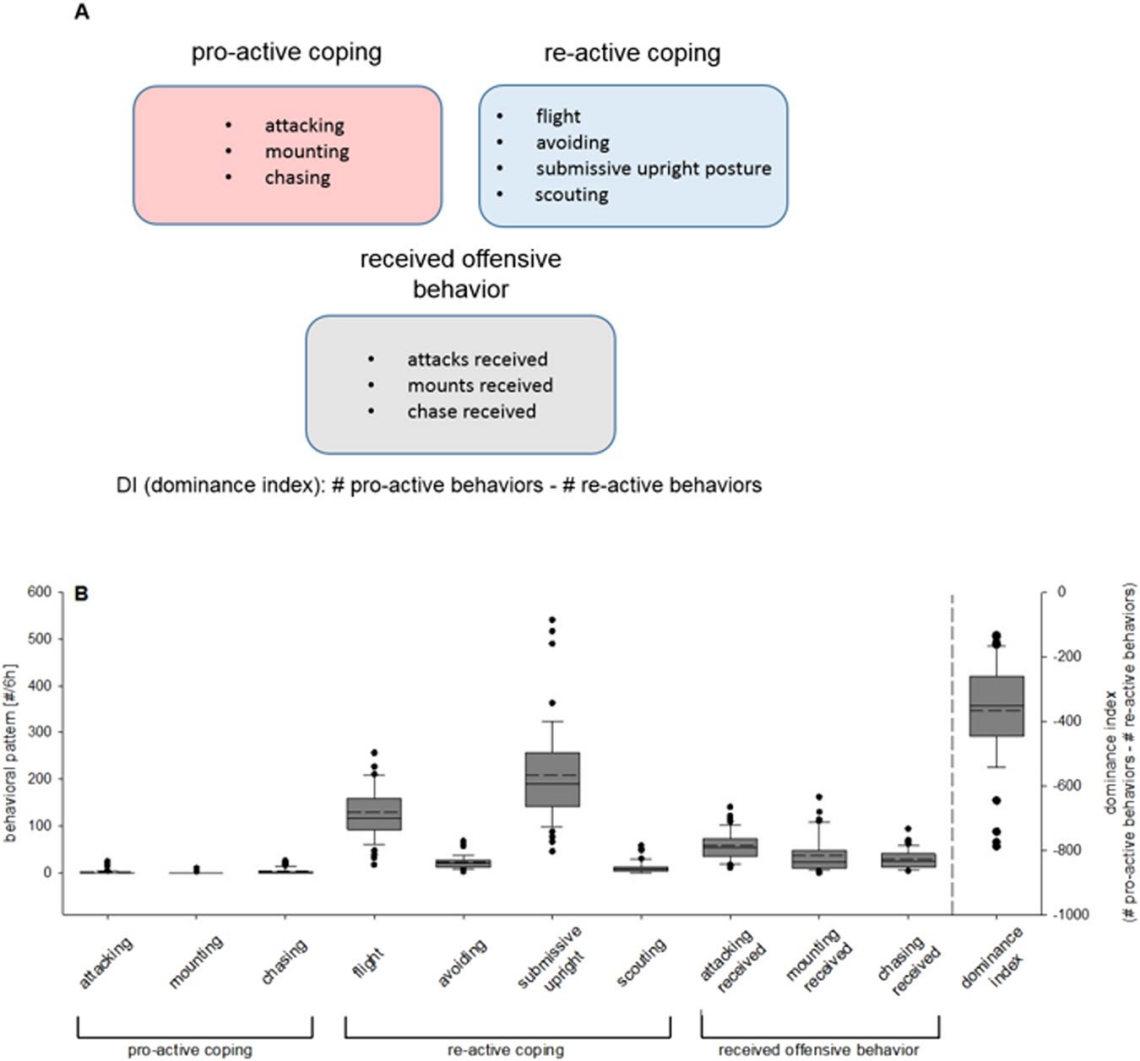
Assessment of individual behavioral coping patterns in chronic subordinate colony housing (CSC) mice. Pro-active, re-active and received offensive behavior shown during CSC exposure were assessed as detailed in (**A**), allowing calculation of a dominance index (DI) for each CSC mouse. Each behavioral pattern was analyzed in CSC mice (n = 46) on days 1, 8 and 15 for one hour in the morning (Supplementary Fig. 2A)  and one hour in the evening (Supplementary Fig. 2B) of the respective days and an overall DI was calculated as in detail shown in (**B**).  CSC.

CSC mice preferentially showed a re-active coping strategy, with flight and submissive upright being the most prominent behaviors (Fig. 1B, Supplementary Fig. 2A,B). However, the individual behaviors as well as the calculated dominance indices (DI) varied considerably among the individuals, providing an optimal prerequisite for correlational analyses between these behavioral elements, the bite score (Supplementary Fig. 1, Supplementary Table 1) and assessed spleen parameters.

### Effects of CSC on typical stress parameters

**Figure 2:**
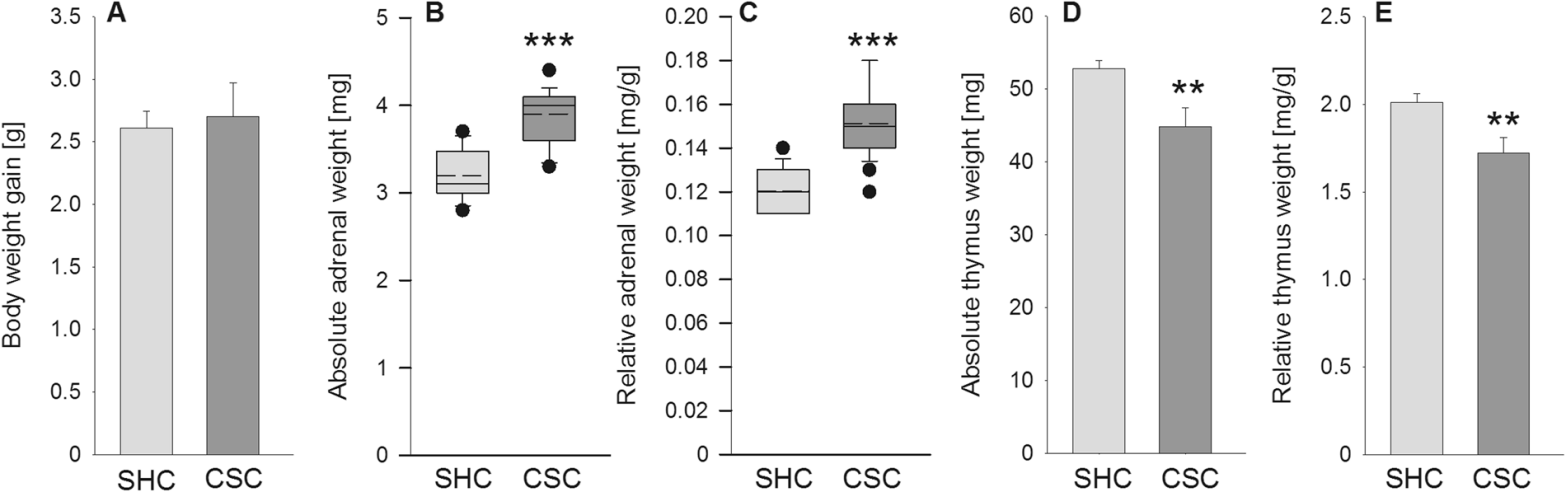
Effects of 19 days of chronic subordinate colony housing (CSC) on physiological parameters. On day 20 of CSC, CSC (n = 23) and respective single-housed control (SHC; n = 24) mice were weighed before decapitation. Following decapitation, adrenal glands as well as thymus were removed, pruned of fat and weighed. Despite an unchanged body weight gain [g] (**A**), CSC mice showed a significant increase in the absolute [mg] (**B**) and relative [mg/g] (**C**) adrenal weight and a significantly decreased absolute [mg] (**D**) and relative [mg/g] (**E**) thymus weight.  SHC;  CSC. **P ≤ 0.01, ***P ≤ 0.001 versus respective SHC.

Statistical analysis revealed no differences in body weight gain (Fig. 2A) between CSC (n = 23) and single housed control (SHC; n = 24) mice. However, 19 days of CSC exposure significantly increased absolute (*P* < 0.001; Fig. 2B) and relative (*P* < 0.001; Fig. 2C) adrenal weight, as well as significantly decreased absolute (*P* = 0.008; Fig. 2D) and relative (*P* = 0.007; Fig. 2E) thymus weight, confirming that the CSC procedure worked and reliably induced a state of chronic psychosocial stress. Identical effects of CSC on adrenal and thymus weight have been reported for the remaining SHC (n = 24) and CSC (n = 23) mice used in this study^30,31^.

### Effects of 19 days of CSC on the spleen

**Figure 3:**
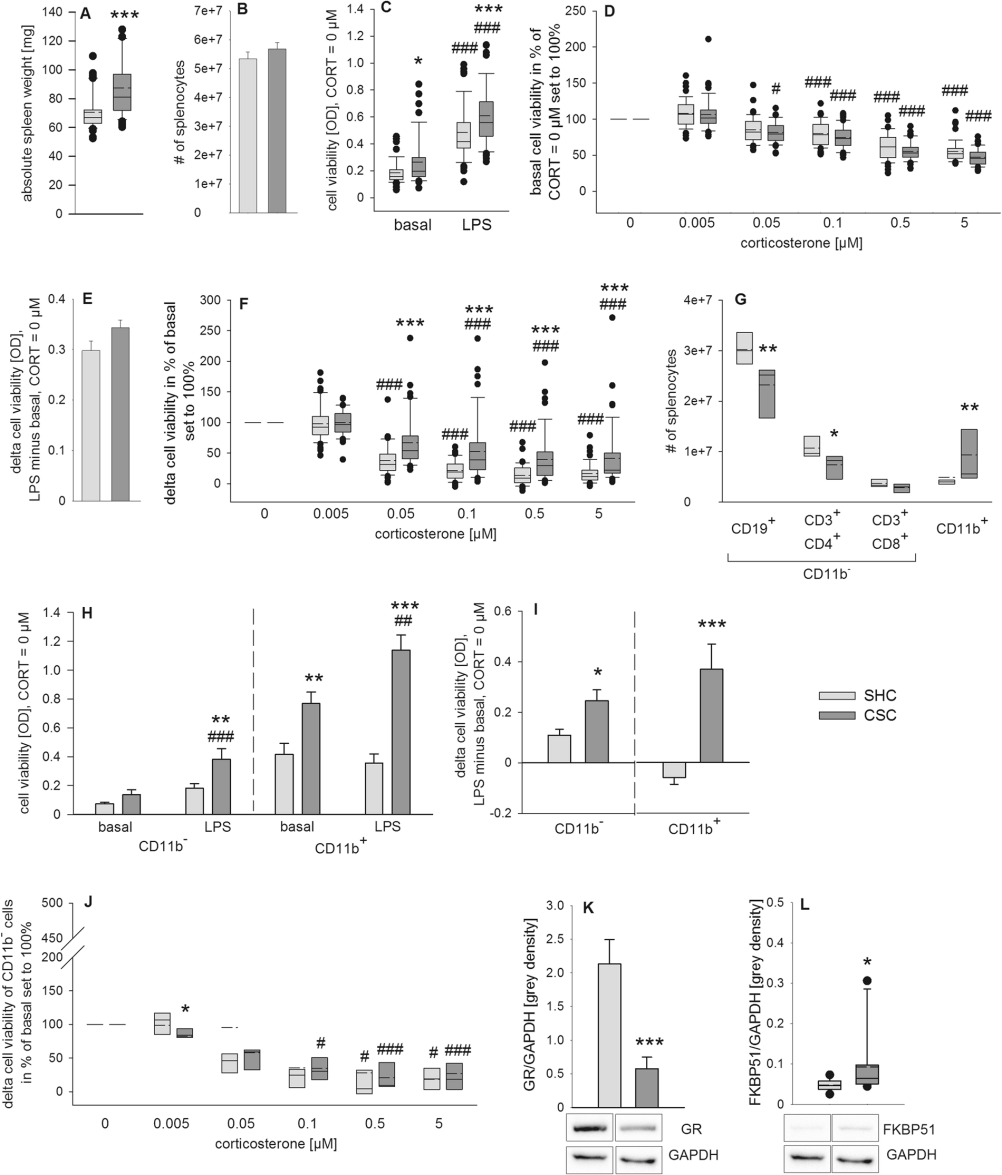
Effects of 19 days of chronic subordinate colony housing (CSC) on the spleen, in the presence and absence of CD11b^+^-cells. Depicted is the absolute spleen weight [mg] (**A**) and the number of splenocytes per spleen (**B**) following 20 days of CSC (n = 48) and single-housing (single housed controls, SHC; n = 48). Splenocytes were cultured without (basal) and with lipopolysaccharide (LPS, 1 µg/ml) in presence of various corticosterone (CORT) concentrations. After 48 h of incubation, cell viability was measured. Depicted is the cell viability [optical density (OD)] under basal and LPS-stimulated conditions in the absence of CORT (**C**) and the basal cell viability (without LPS) in the presence of different CORT concentrations (CORT = 0 µM was set to 100%; **D**). Delta cell viability of LPS-stimulated minus respective basal wells at CORT = 0 µM is depicted in (**E**). Shown is further the delta cell viability [OD] (LPS-stimulated minus respective basal wells) at various CORT concentrations in percent of respective values at CORT = 0 µM (set to 100%; **F**). Depicted are further the absolute numbers of CD19^+^, CD3^+^CD4^+^, CD3^+^CD8^+^ and CD11b^+^ cells within total viable splenocytes (**G**) of another set of SHC (n = 8) and CSC (n = 8) mice. CD11b^+^ as well as CD11b^−^ cells from these mice were cultured without (basal) and with LPS (1 µg/µl) in absence or presence of various CORT concentrations. Depicted is the cell viability under basal and LPS-stimulated conditions (**H**) as well as delta cell viability of LPS-stimulated minus respective basal wells (**I**) of CD11b^−^ and CD11b^+^ splenocytes. Shown is further the delta cell viability (LPS-stimulated minus respective basal wells) of CD11b^−^ splenocytes at various CORT concentrations in percent of respective values at CORT = 0 µM (set to 100%; **J**). A third set of SHC (n = 11) and CSC (n = 10) mice was employed to determine relative protein expression of glucocorticoid receptor (GR; **K**) and FK506 binding protein 51 (FKBP51; **L**) [grey density] on day 20 of CSC using western blotting, all normalized to the loading control glyceraldehyde 3-phosphate dehydrogenase (GAPDH). Representative images of bands detected for GR (~86 kDa) and FKBP51 (~51 kDa) and respective loading control GAPDH (~36 kDa) are shown for SHC and CSC mice below each subfigure.  SHC;  CSC. *P ≤ 0.05, **P ≤ 0.01, ***P ≤ 0.001 versus respective SHC; ^#^ *P* ≤ 0.05, ^##^ *P* ≤ 0.01, ^###^ *P* ≤ 0.001 versus respective basal (CORT = 0 µM) values.

Statistical analysis revealed an increase in absolute spleen weight (*P* < 0.001; Fig. 3A) in CSC compared with SHC mice while the number of isolated splenocytes was comparable between both groups (Fig. 3B). When CORT was absent (0 µM CORT), stimulation of isolated splenocytes with LPS for 48 h (Fig. 3C) was shown to increase the cell viability in SHC (*P* < 0.001) as well as in CSC mice (*P* < 0.001) compared with unstimulated (basal) conditions. Moreover, CSC mice showed higher spleen cell viability under basal (*P* = 0.011) as well as under LPS-stimulated conditions (*P* = 0.001) compared with respective SHC mice (Fig. 3C), in line with a strong tendency towards an increased delta cell viability (LPS minus basal, 0 µM CORT; *P* = 0.056; Fig. 3E) in CSC versus SHC mice. Under unstimulated (basal) conditions, statistical analysis revealed a significant main effect of factor CORT in SHC (Χ² (5, 48) = 192.047, *P* < 0.001) and CSC mice (X² (5, 46) = 203.128; *P* < 0.001; Fig. 3D). Cell viability in the presence of 0.05 µM (SHC: *P = *0.058; CSC: *P* = 0.012), 0.1 µM (SHC and CSC: *P* < 0.001), 0.5 µM (SHC and CSC: *P* < 0.001) and 5 µM (SHC and CSC: *P* < 0.001) CORT was significantly lower in both SHC and CSC mice compared with the respective 0 µM CORT condition (set to 100%). Statistical analysis further revealed a significant main effect of factor CORT in SHC (Χ² (5, 48) = 188.976, *P* < 0.001) and CSC mice (X² (5, 46) = 153.369; *P* < 0.001) on delta cell viability (LPS minus basal) during *in vitro* stimulation of isolated spleen cells with different CORT concentrations (Fig. 3F). In detail, delta cell viability in the presence of 0.1 µM (SHC and CSC: *P* < 0.001), 0.5 µM (SHC and CSC: *P* < 0.001) and 5 µM CORT (SHC and CSC: *P* < 0.001) was significantly lower in both SHC and CSC mice compared with the respective 0 µM CORT condition (set to 100%). However, delta cell viability in the presence of 0.05 µM CORT was lower compared with the 0 µM CORT condition (set to 100%) in SHC (*P* < 0.001), but not in CSC, mice. Importantly, separate Mann-Whitney-U tests revealed a significantly higher delta cell viability in CSC compared with respective SHC mice at 0.05 µM (*P* < 0.001), 0.1 µM (*P* < 0.001), 0.5 µM (*P* < 0.001) and 5 µM (*P* < 0.001) CORT.

Statistical analysis further revealed a significant decrease in CD19^+^ cells (*P* = 0.009; Fig. 3G) as well as in CD3^+^CD4^+^ cells (*P* = 0.021; Fig. 3G) in CSC compared with SHC mice, while the number of CD3^+^CD8^+^ cells remained unchanged (Fig. 3G). The absolute number of CD11b^+^ cells was significantly increased following CSC exposure (*P* = 0.006; Fig. 3G). Cell viability of CD11b^−^ splenocytes under unstimulated (basal) and LPS-stimulated conditions was found to be dependent on factor CSC (F_1,26_ = 9.678; *P* = 0.004) and factor stimulus (F_1,26_ = 17.581; *P* < 0.001, Fig. 3H left panel) when CORT was absent (0 µM CORT). Post hoc Bonferroni pairwise comparisons revealed an effect of LPS on cell viability of splenocytes of CSC mice (*P* < 0.001) while there was only a trend in splenocytes of SHC mice (*P* = 0.070) compared with basal values (Fig. 3H left panel). Under LPS-stimulated conditions, cell viability was higher in CSC compared with SHC mice (*P* = 0.003). Cell viability of CD11b^+^ splenocytes under unstimulated (basal) and LPS stimulated conditions was found to be dependent on the factor CSC (F_1,24_ = 55.770; *P* < 0.001) and on the interaction of factors stimulus and CSC (F_1,24_ = 6.284; *P* = 0.019; Fig. 3H right panel) when CORT was absent (0 µM CORT). Post hoc Bonferroni pairwise comparisons revealed an effect of LPS on cell viability of CSC, but not SHC, splenocytes (*P* = 0.004) compared with respective basal values (Fig. 3H right panel). Furthermore, cell viability was increased in CSC compared with SHC mice under basal (*P* = 0.002) and LPS (*P* < 0.001) conditions. Moreover, delta cell viability (LPS minus basal, 0 µM CORT) was increased in CSC versus SHC mice when CD11b^−^ (*P* = 0.012) or CD11b^+^ (*P* < 0.001) splenocytes were plated (Fig. 3I). Statistical analysis further revealed a significant main effect of factor CORT in SHC (Χ² (5, 8) = 20.071, *P* = 0.001) and CSC (X² (5, 7) = 29.122; *P* < 0.001) mice on delta cell viability (LPS minus basal) during *in vitro* stimulation of isolated CD11b^−^ spleen cells with different CORT concentrations (Fig. 3J). In detail, delta cell viability in the presence of 0.1 µM (SHC: *P* = 0.075; CSC: *P = *0.015), 0.5 µM (SHC: *P* = 0.032; CSC: *P* = 0.001) and 5 µM CORT (SHC: *P* = 0.049; CSC: *P* = 0.001) was by trend or significantly lower in both SHC and CSC mice compared with the respective 0 µM CORT condition (set to 100%). Separate Mann-Whitney-U tests revealed a significantly lower cell viability in CSC compared with SHC mice only at 0.005 µM CORT condition (*P* = 0.049).

### Potential molecular mechanisms of CSC-induced GC resistance

Statistical analysis revealed a significant decrease of GR (Fig. 3K; *P* = 0.001) and a significant increase of FK506 binding protein 51 (FKBP51) (Fig. 3L; *P* = 0.017) protein expression in isolated splenocytes of CSC compared with SHC mice. The bite score of the CSC mice in this subset of experimental animals was 13.70 ± 3.57.

### Correlational analyses

**Figure 4:**
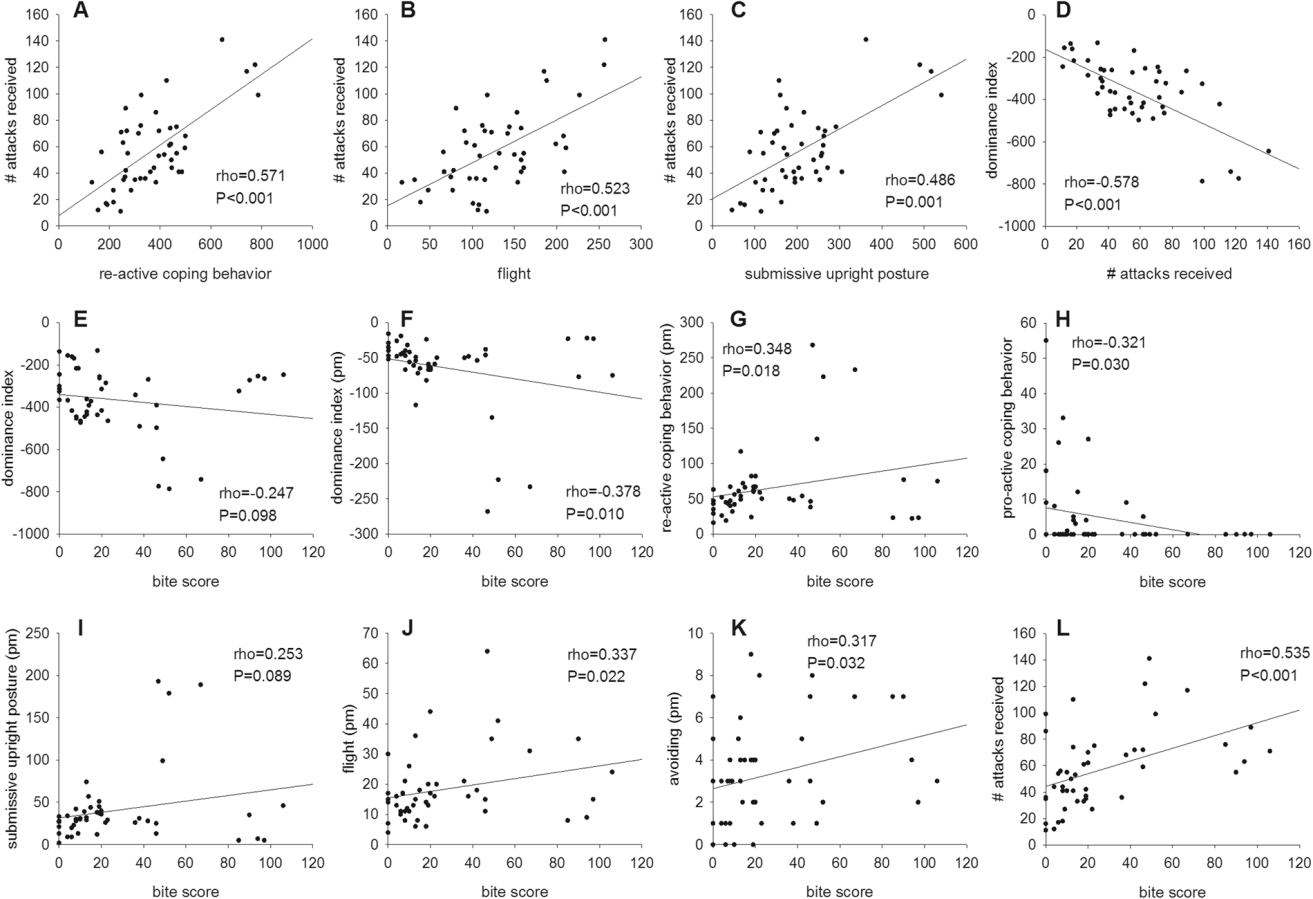
Correlation analyses - Part I. Depicted are significant correlations between individual behavioral coping patterns shown in the morning (am) and/or in the evening (pm) on days 1, 8 and 15 with the bite scores in all CSC (n = 46) mice. The number of attacks received was positively correlated with the re-active coping behavior (**A**), flight (**B**), as well as with submissive upright posture (**C**). A negative correlation was found between the dominance index (DI) and the number of attacks received (**D**), as well as the bite score (**E**). The DI in the evening also correlated negatively with the bite score (**F**). Furthermore, the bite score was positively correlated with re-active coping behavior of CSC mice in the evening (**G**), whereas it correlated negatively with the amount of pro-active behavior (**H**). In detail, the bite score showed a positive correlation with submissive upright posture (**I**), flight (**J**), avoiding (**K**), all in the evening, as well as the total number of attacks received (**L**).

The detailed analysis of pro-active (attacking, chasing, mounting), re-active (flight, avoiding, submissive upright posture, scouting), and received offensive (attacks received, mounts received, chase received) behaviors of all CSC mice in the morning (1000 h–1100 h; am) and in the evening (1700 h–1800 h; pm) on days 1, 8, and 15 revealed a positive correlation between re-active behaviors and the number of attacks received (rho = 0.571; *P* < 0.001; Fig. 4A). In detail, flight (am: rho = 0.541; *P* < 0.001; Supplementary Fig. 3A; pm: rho = 0.351; *P* = 0.017; Supplementary Fig. 3B; am + pm: rho = 0.523; *P* < 0.001, Fig. 4B) as well as submissive upright (am: rho = 0.516; *P* < 0.001; Supplementary Fig. 3C; pm: rho = 0.446; *P* = 0.002; Supplementary Fig. 3D; am + pm: rho = 0.486; *P* = 0.001; Fig. 4C) correlated positively with the numbers of attacks received at the respective time point of assessment. In line, the dominance index (DI), which represents the number of pro-active minus the number of re-active behaviors, correlated negatively with the number of attacks received (am: rho = −0.592; *P* < 0.001; Supplementary Fig. 3E; pm: rho = −0.446; *P* = 0.002; Supplementary Fig. 3F; am + pm: rho = −0.578; *P* < 0.001; Fig. 4D) at the respective time point of assessment.

Given that there is further a negative correlation of the DI with the bite score (am + pm: rho = −0.247, *P* = 0.098; Fig. 4E; pm: rho = −0.378, *P* = 0.010; Fig. 4F), while re-active (pm: rho = 0.348; *P* = 0.018; Fig. 4G) behavior in general as well as submissive upright (pm: rho = 0.253; *P* = 0.089; Fig. 4I), flight (pm: rho = 0.337; *P* = 0.022; Fig. 4J) and avoiding (pm: rho = 0.317; *P* = 0.032; Fig. 4K) correlating significantly or at least by trend positively with the bite score and pro-active coping behavior correlating negatively with the bite score (rho = −0.321; *P* = 0.030; Fig. 4H), this strongly suggests that a re-active coping strategy during CSC exposure not only increases the risk of getting attacked by the resident, but also of getting injured. In line with this hypothesis, the number of attacks received was positively correlated with the bite score (am: rho = 0.526; *P* < 0.001; Supplementary Fig. 3G; am + pm: rho = 0.535; *P* < 0.001; Fig. 4L).

**Figure 5:**
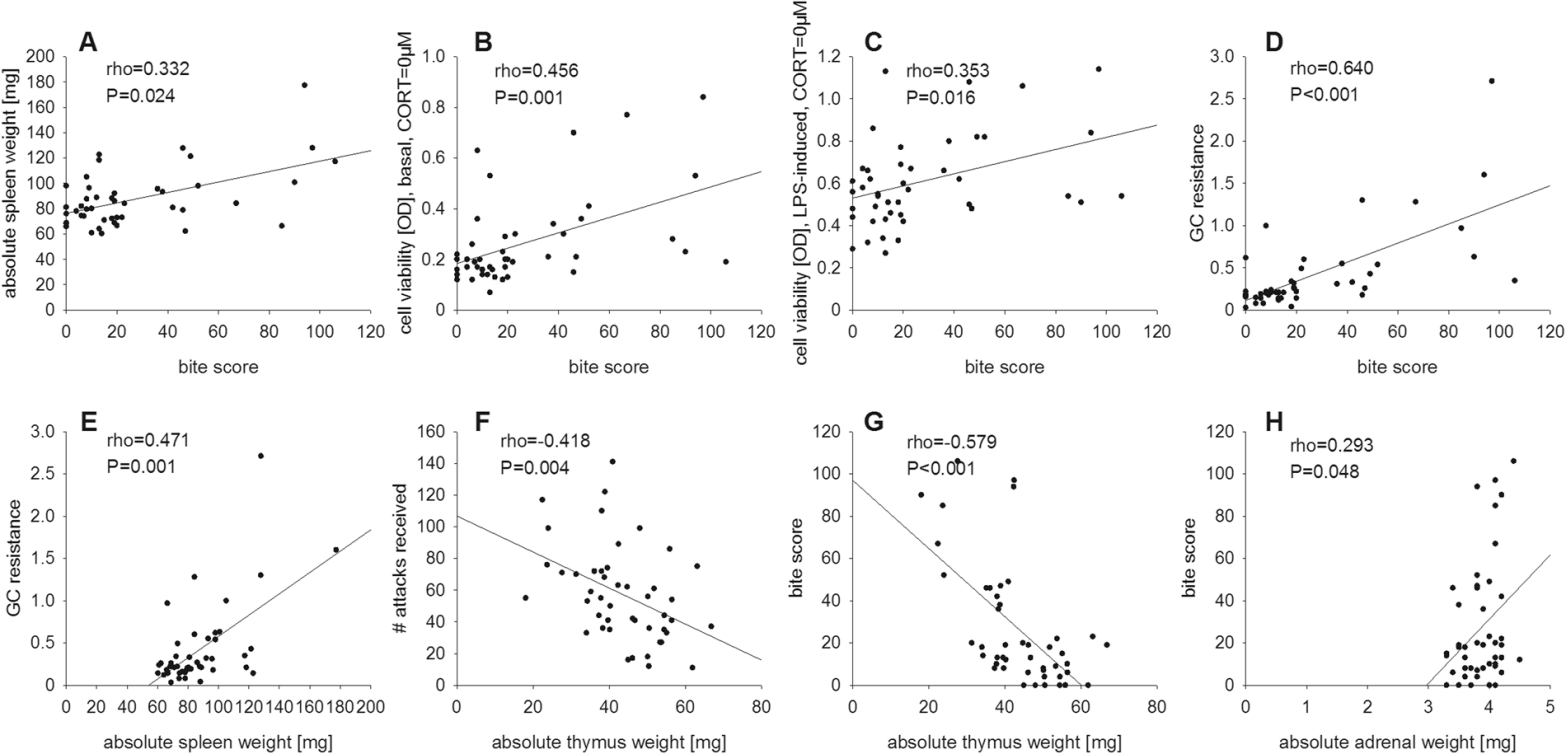
Correlation analyses – Part II. Depicted are significant correlations between individual behavioral coping patterns shown in the morning and in the evening on days 1, 8 and 15 with the bite scores as well as physiological parameters in all CSC (n = 46) mice. The absolute spleen weight (**A**), the cell viability of isolated splenocytes under basal (**B**) and LPS-stimulated (**C**) conditions (CORT = 0 µM), as well as the splenic glucocorticoid (GC) resistance (**D**) correlated positively with the established bite score of each CSC mouse. The developed GC resistance (**E**) was also positively correlated with the absolute spleen weight. The number of attacks received (**F**) and bite score (**G**) correlated negatively with the absolute thymus weight, whereas the absolute adrenal weight was positively correlated with the bite score (**H**).

Considering the positive correlation of the bite score and the absolute spleen weight (rho = 0.332; *P* = 0.024; Fig. 5A), and the development of splenic GC resistance (rho = 0.640; *P* < 0.001; Fig. 5D), our data suggest that being bitten during social confrontations causes splenic immune activation and GC resistance, probably to adequately fight against invading pathogens. Of note in this context, the cell viability under basal (rho = 0.456; *P* = 0.001; Fig. 5B) and LPS (rho = 0.353; *P* = 0.016; Fig. 5C) conditions at 0 µM CORT also correlated positively with the bite score and the development of splenic GC resistance was positively correlated with absolute spleen weight (rho = 0.471; *P* = 0.001; Fig. 5E).

Finally, attacks received (rho = −0.418; *P* = 0.004; Fig. 5F) and bite score (rho = −0.579, *P* < 0.001; Fig. 5G) correlated negatively with the absolute thymus weight, while absolute adrenal weight correlated positively with the bite score (rho = 0.293; *P* = 0.048; Fig. 5H), indicating that being attacked (thymus) and getting bitten (adrenal and thymus) strongly influence the perceived severity of a social stressor by an individual.

## Discussion

In the present study, we show that 19 days of CSC cause splenomegaly and GC resistance in *in vitro* LPS-stimulated spleen cells, effects that were mediated by CD11b^+^ cells. Together with a decreased splenic GR and increased FKBP51 expression, suggesting a reduced receptor availability and compromised nuclear translocation, these findings are in line with previously reported effects of SDR exposure. We further extend these findings by showing that in the absence of *in vitro* CORT exposure, CSC increased *in vitro* cell viability under basal and LPS conditions as well as the delta response to LPS (by trend; LPS – basal), effects that were exaggerated but not solely mediated by CD11b^+^ cells. Importantly, detailed behavioral analyses during CSC exposure, in combination with establishing a novel bite score, which considers the dermal and subdermal area and intensity of bite wounds, allowed us to extend the existing knowledge on the link between different stress coping strategies and being wounded and, in turn, developing splenic immune activation and GC resistance during social stressor exposure. In summary, our data indicate that the mechanisms underlying the skin injury-dependent and CD11b^+^ cell-mediated splenic immune activation and GC resistance following social stress in male mice are paradigm independent and, thus, of general importance for paradigms allowing direct physical interaction and, perhaps, for humans facing physical trauma in the context of psychosocial stress. They further reveal that there is a clear link between bite wounds and re-active stress coping style, characterized by behaviors like submissive upright, scouting, flight and avoiding. Moreover, our data indicate that chronic social stress without the occurrence of any skin injuries, in a CD11b^+^ cell independent manner, promotes splenic immune activation, likely contributing to systemic low grade inflammation reported following many chronic stressors in humans and laboratory animals.

In a series of experiments Sheridan and colleagues showed that SDR, but not immobilization, promotes splenomegaly and GC resistance in *in vitro* Con A- or LPS-stimulated spleen cells^16–18^, that this effect is mediated by CD11b^+^ cells migrating from the bone marrow to the spleen^20,21^, and that GC resistance in these cells is likely mediated by decreased GR expression as well as compromised GR nuclear translocation^22,23^. In line with the above mentioned and our previous data^27,32,33^, CSC compared to SHC mice in the present study showed an increased adrenal and a decreased thymus weight, an unchanged body weight gain, splenomegaly, increased percentage of CD11b^+^ spleen cell counts, and GC resistance in *in vitro* LPS-stimulated spleen cells, an effect that was absent if CD11b^+^ splenocytes were depleted. Development of splenomegaly, despite a comparable number of splenocytes between SHC and CSC mice, could be due to an altered composition of splenocytes, with an increase in larger CD11b^+^ cells and a decrease in smaller CD3^+^ and CD19^+^ cells^34^. Although a lack of more mechanistic details clearly poses a limitation of the current study, a general reduction of GR combined with a compromised GR nuclear translocation are at least likely to play a role in CSC-induced spleen effects, again in line with previous SDR data. This was suggested by reduced GR protein expression, while FKBP51 protein expression was increased, both assessed in isolated splenocytes of CSC versus SHC mice. FKBP51 is a co-chaperone of one of the most prominent chaperones bound to the GR, the heat shock protein 90, together regulating GR functionality/sensitivity. An increase in FKBP51 expression, for instance, is induced by GC via an ultra-short feedback loop, resulting in a reduction of sensitivity and in turn a reduction of nuclear translocation of the GR^35^. Of note, as shown earlier by our group^33^, the percentage of splenic B cells (CD19^+^) and T helper cells (CD3^+^CD4^+^) was lower in CSC versus SHC mice, suggesting that the adaptive, in contrast to the innate, immune system, at least in the spleen, is inhibited.

In addition, in the absence of *ex vivo* CORT, splenocytes from CSC versus SHC mice showed an increased cell viability when cultured *in vitro* under basal or LPS conditions and showed a by trend increased delta response in cell viability to LPS, effects that were more pronounced in isolated CD11b^+^ cells, but also detectable in splenocytes depleted of CD11b^+^ cells (=CD11b^−^ cells). This indicates that, in contrast to splenic GC resistance, enhanced spleen cell (re) activity following CSC is mediated by various subtypes of splenocytes, including CD11b^+^ cells, in a wounding dependent and independent manner. Although, at least to our knowledge, only the exaggerated delta response in cell viability and IL-6 secretion to LPS (LPS – basal values at 0 µM CORT) has been reported following SDR^16,17,20^, it is very likely that also the *in vitro* cell viability during basal and LPS conditions is increased following SDR, given the high consistency of all other so far reported spleen effects of SDR and CSC exposure and the increased *in vitro* microbicidal activity reported for CD11b^+^ splenocytes from SDR mice^36^. Intriguingly, and this is again consistent with findings reported following SDR^16^, splenic GC resistance only was detectable when the cells were stimulated with LPS *in vitro*; GC sensitivity of splenocytes not exposed to LPS was comparable between CSC and SHC mice, neglecting the reduced cell viability in CSC versus SHC mice at only the 0.005 µM CORT condition. Together with our previous findings^27^ and the fact that GC resistance is absent following immobilization stress^18^ the data of the current study so far suggest that splenic immune activation, due to enhanced basal and LPS-induced spleen cell (re)activity of both CD11b^+^ and CD11b^−^ splenocytes in combination with GC resistance of specifically CD11b^+^ splenocytes, represents a general and paradigm independent consequence of chronic psychosocial stress.

However, the absence of GC resistance in splenocytes from mice exposed to a mild social stressor without direct physical interaction of the opponents and, thus, without receiving bite wounds suggests that wounding plays a central role in social stress-induced GC resistance^24^. In support, Sheridan and colleagues showed in one of their earlier SDR studies that GC resistance of LPS-stimulated spleen cells only develops in wounded (deep lesions of the skin, genital area, or tail), but not non-wounded (minor superficial scratches or no visible wounds) SDR mice. Thus, instead of the social character of a certain social stressor per se, the resulting skin injuries seem to be the critical factor promoting splenic immune activation and GC resistance following social stress^19^. In the current study we confirm and extend these findings by showing in another social stress paradigm allowing direct physical interaction that absolute spleen weight, *in vitro* spleen cell viability during basal and LPS conditions, delta spleen cell response to LPS and development of splenic GC resistance following LPS challenge in CSC mice correlated positively with the severity of bite wounds. The latter was assessed by a specifically developed bite score, considering in detail dermal and subdermal area and intensity of bite wounds. This clearly indicates that the link between bite wounds and splenic immune activation or GC resistance is not a binary but a rather continuous one.

Importantly, given that SDR increases spleen weight and the number of splenic CD11b^+^ cells also under germ-free conditions^36^ – GC resistance of LPS-stimulated splenocytes has not been tested yet – it is further likely that social stress effects on at least these spleen parameters are solely due to the severity of wounding and not dependent on skin or gut bacteria translocating to the spleen. As splenic *in vitro* GC resistance following CSC and SDR^16^ was further only detectable after adding LPS to the culture medium, it is likely that *in vivo* GC resistance following social stress and wounding, in contrast to CD11b^+^ invasion and splenomegaly, requires bacterial signals in the spleen, either from the skin or the gut, resulting in toll-like receptor (TLR)−4 activation in CD11b^+^ cells. Given that SDR exposure^37^ has been shown to cause bacterial translocation of cutaneous and/or gastrointestinal microflora to inguinal and/or mesenteric lymph nodes, but not to the spleen, it seems likely that CD11b^+^ cells in the spleen need to get in direct contact with bacterial stimuli to develop GC resistance. Of note, bacterial translocation to colonic tissue, but not to the mesenteric lymph nodes has also been shown in CSC mice^38^, suggesting a lack of bacterial signals derived from the gut microbiota in the spleen of CSC mice. Of note, given the central role of wounding in social stress-associated GC resistance, it remains to be investigated in detail whether the lack of an effect of immobilization stress on GC resistance in *in vitro* Con A-stimulated spleen cells^18^ is due to the absence of wounding or because immobilization stress does not hold a social component. Translocation of cutaneous and gastrointestinal microflora to inguinal and mesenteric lymph nodes and the liver has, however, been reported following immobilization stress^37^. Of special note in terms of the translational value of our data into the human context, in which social stress only in certain occupational groups, i.e. policemen and soldiers on active duty, is likely to go along with skin injuries, is the fact that depletion of bite wound-induced CD11b^+^ cells from CSC splenocytes did not completely prevent splenic immune activation, pointing towards a mechanism contributing to systemic low grade inflammation reported following many chronic stressors in a wounding independent manner. Moreover, our preliminary mouse data indicate that abdominal surgery prior to chronic psychosocial stress can facilitate the development of splenic GC resistance independent of bite wounds received during direct physical contact, suggesting that planned or unplanned surgery in humans in combination with chronic stress is likely to promote splenic GC resistance and, thus, the risk for surgical complications related to an overshooting immune response.

But which behavioral stress coping profile favors injuries and, thus, splenic immune activation and GR resistance during social confrontations? In general, active coping behavior, characterized by higher aggression, attempts to counter an aggressor’s attacks, longer submission latencies and sympathetic nervous system activation, is considered to be more effective in moderating the stress associated with social defeat than passive, i.e. submissive coping behavior associated with HPA-axis activation^39,40^. For instance, intruder rats with a longer submission latency to the resident recovered faster from disturbances in lymphocyte numbers and body weight than intruders submitting more quickly^41^. In addition, intruder rats attempting (active) versus non-attempting (passive) to counter the resident’s attacks showed less pronounced disturbances in diurnal heart rate, body temperature and locomotor activity rhythms^42^. Moreover, Walker and colleagues show that active coping behavior, indicated by engagement in a large number of fights and/or the frequent use of physical structures to block the resident’s approach, is associated with both a smaller adrenocortical response and a lower level of neural activation in forebrain regions such as the medial prefrontal cortex and the amygdala following social defeat^39^. Given that GC resistance has been shown to only develop in wounded SDR mice, the positive correlation found between GC resistance and the duration of submissive behavior during the first SDR cycle^19^ suggests that passive, i.e. submissive, stress coping also in the SDR paradigm seems to be mal-adaptive and result in more wounding and, consequently, splenic immune activation and GC resistance. Detailed behavioral analysis of pro-active, i.e. attacking, mounting and chasing, and re-active, i.e. flight, avoiding, submissive upright and scouting, coping during CSC exposure in the current study supports and extends this hypothesis. While submissive upright was positively correlated with the number of attacks received, the same was true for flight and avoiding as well as total re-active behavior, suggesting that the risk of being attacked is independent from the type of re-active coping strategy. In support, the DI, which represents the number of pro-active minus the number of re-active behaviors, correlated negatively with the number of attacks received. As the number of attacks received as well as the re-active coping, in particular flight, avoiding and submissive upright (by trend; rho = 0.253; *P* = 0.089) further correlated positively with the bite score, this type of coping style seems to increase the risk of being wounded and, consequently, of developing splenic immune activation and GC resistance during social confrontations, although causality still needs to be addressed in future studies. However, the lack of a positive correlation between re-active coping and GC resistance either does not support this conclusion or indicates that the bite score is further dependent on behaviors not assessed in the current study, representing another limitation of the current study. Of note, pro-active coping and the DI correlated negatively with the bite score, which is in line with our recent study showing that a shift towards more pro-active stress coping during CSC, induced by repeated subcutaneous (s.c.) preimmunization with a heat-killed preparation of *Mycobacterium vaccae* (National Collection of Type Cultures (NCTC) 11659), an abundant soil saprophyte with immunoregulatory properties, promotes stress-resilience and ameliorates stress/trauma-induced anxiety and colitis^10^.

In summary, this study supports the hypothesis that the mechanisms underlying chronic psychosocial stress-induced splenic immune activation and GC resistance are stress paradigm independent and to a large extent mediated by spleen-invading CD11b^+^ cells. Moreover, social stress-associated wounding, instead of the social character of the stressor per se, seems to be the critical trigger of these CD11b^+^ cell-mediated spleen changes, as suggested by the positive correlation between the severity of bite wounds and the dimensions of basal and LPS-induced spleen cell activation and splenic GC resistance in CSC mice. Although causality needs to be addressed in detail in future studies, our data further suggest that social stress-associated wounding and CD11b^+^-mediated splenic immune activation and GR resistance, are promoted by behaviors like flight, avoiding, submissive upright and scouting, collectively termed as re-active stress coping. Importantly, in addition to these wounding dependent and CD11b^+^ cell-mediated spleen effects, which only in certain occupational groups, during planned and unplanned surgeries, or in the context of physical trauma (e.g. accident) might become relevant in the human context, social stressors also in an injury and CD11b^+^ cell independent manner promote spleen cell activation, but not GC resistance.

## Material and Methods

### Animals

Male C57BL/6 N mice (Charles River, Sulzfeld, Germany) weighing 19–22 g (experimental mice) were individually housed in standard polycarbonate mouse cages (16 × 22 × 14 cm) for one week before the CSC paradigm started. Male CD-1 mice (weighing 30–35 g; Charles River, Sulzfeld, Germany) were used as dominant animals. All mice were kept under standard laboratory conditions (12 h light/12 h dark cycle, lights on at 0600 h, 22 °C, 60% humidity) and had free access to tap water and standard mouse diet. All experimental protocols were approved by the Committee on Animal Health and Care of the local government, and conformed to international guidelines on the ethical use of animals. All efforts were made to minimize the number of animals used and their suffering.

### Experimental procedures

All experimental mice were either chronically stressed by 19-day exposure to the CSC paradigm or single-housed for control (SHC). In one set of SHC (n = 48) and CSC (n = 46) mice, spleen parameters, in detail spleen weight, number of splenocytes and *in vitro* GC sensitivity of isolated splenocytes were analysed. Moreover, a detailed behavioral analysis and evaluation of bite wounds was done in the CSC mice. In a subset of these animals (SHC: n = 24; CSC: n = 23) general physiological parameters like body weight gain, adrenal and thymus weight as well as molecular parameters like GR and FKBP51 protein expression in splenocytes (SHC: n = 11; CSC: n = 10) were analysed. Data for adrenal and thymus weights have already been reported for the remaining subset of SHC (n = 24) and CSC (n = 23) mice^30,31^. Another set of SHC (n = 8) and CSC (n = 8) was used for flow cytometric analysis of splenocytes and subsequent depletion of CD11b^+^ splenocytes and analysis of *in vitro* GC sensitivity.

### Chronic subordinate colony housing (CSC)

The CSC paradigm was conducted as described previously^3,27,32,43^. Briefly, four experimental CSC mice were housed together with a dominant male mouse for 19 consecutive days, in order to induce a chronic stressful situation. Before the CSC procedure, the future dominant males were tested for their aggressive behavior and mice that injured their opponents by excessive aggression were excluded. Notably, the number of bite wounds received by the residents could thereby be reduced, but not totally prevented. To avoid habituation, each dominant male was replaced by a novel dominant male at days 8 and 15 of the CSC procedure. To confirm the intended dominant/subordinate hierarchy within each colony and to analyse the individual stress coping behavior, mice were always videotaped during the first hour after putting them together (1000 h–1100 h am) on days 1, 8 and 15 and in the respective evening hours (1700 h–1800 h pm). SHC mice remained undisturbed in their home cages except for change of bedding once a week. In a previous study we convincingly demonstrated that single housing is the adequate control group for the CSC paradigm, as group housing itself was shown to be stressful and to affect parameters assessed routinely in studies employing the CSC paradigm^44^.

### Behavioral analysis during CSC exposure

To assess possible differences in the behavioral stress coping strategy between the individual CSC mice (pro-active vs. re-active coping) during 19 days of CSC exposure, mice of each CSC colony were videotaped for 1 h in the morning (1000 h–1100 h am) and in the evening (1700 h–1800 h pm) on days 1, 8 and 15. As depicted in Fig. 1A and described in Supplementary Table 1, their agonistic behavior was individually analyzed in terms of pro-active (subdominant: attacking, chasing, and mounting) and re-active (submissive upright posture, scouting, flight and avoiding) coping and received offensive behavior (attacks, mounts and chase received). All different behaviors were scored individually for each CSC mouse and a dominance index (DI; number of pro-active minus number of re-active behaviors) was calculated.

### Determination of adrenal, thymus and spleen weight

After decapitation under CO_2_ anaesthesia in the morning (between 0600 h and 1000 h) of day 20, the adrenals, thymus and spleen of each mouse was removed, pruned of fat and weighed. Spleens were stored in ice-cold Hanks’ balanced salt solution (HBSS) until cells were isolated.

### Measurement of GC sensitivity/resistance of isolated splenocytes

Each spleen was weighed and splenocytes were isolated as previously described^27^. Briefly, spleens were homogenized to obtain single-cell suspensions. Erythrocytes were removed by adding lysis buffer (155 mM NH_4_Cl, 10 mM KHCO_3_, 10 mM EDTA) for 2 min followed by addition of HBSS/10% heat-inactivated fetal calf serum (FCS). Following filtration of the cell suspension through a 70 µm nylon cell strainer and one washing step, the number of isolated splenocytes was assessed using a cell counter (TC-20, Bio-Rad Laboratories, München, Germany). In the second set of SHC and CSC mice, an aliquot of 1 × 10^6^ cells of SHC and CSC mice was set aside for flow cytometry analysis. Final cell concentration was adjusted to 2.5 × 10^6^ cells/ml in RPMI containing 10% FCS, 50 U/ml penicillin and 50 µg/ml Streptomycin. Cell suspensions were stimulated with LPS (*Escherichia coli* O111:B4; final concentration: 1 µg/ml; Sigma-Aldrich, Deisenhofen, Germany) or remained untreated to assess background activity. For determination of GC sensitivity/resistance, unstimulated and LPS-stimulated cells were treated with various CORT (Sigma-Aldrich, Deisenhofen, Germany) concentrations (final concentration: 0, 0.005, 0.05, 0.1, 0.5 and 5 µM respectively), diluted in 95% ethanol. Cells were stimulated in flat-bottom, 96-well plates at a volume of 100 µl/well and were incubated for 48 h (37 °C, 5% CO_2_). After 48 h, cell viability was measured via a commercially available colorimetric assay (CellTiter 96 Aqueous One Solution Assay, Promega, Madison, WI). Living cells convert the containing formazan to a red dye, which can be measured by the use of an enzyme-linked immunosorbent assay (ELISA) plate reader (Fluostar Optima, BMG Labtech, Offenburg, Germany) at 450 nm. The absorbance (optical density (OD)) of wells with unstimulated cells for a given treatment was subtracted from the corresponding LPS-stimulated well. A low cell viability at increasing CORT concentrations is indicating a high GC sensitivity and a low GC resistance. For correlational analyses cell viability OD values at 5 µM CORT were used to indicate the extent of GC resistance (high OD means high GC resistance, low OD means low GC resistance).

### Splenocytes CD11b depletion

Splenocytes were obtained as described above. Aliquots of splenocytes of SHC and CSC mice were set aside for the GC sensitivity assay and for the flow cytometric analysis. CD11b^+^ cells from the spleen were positively selected according to the manufacture’s protocol using the CD11b MicroBeat Kit (Miltenyi Biotech, Bergisch Gladbach, Germany). In brief, 1 × 10^7^ cells were incubated with 10 µl anti-CD11b microbeads for 15 min at 4 °C. Cells were washed and loaded on pre-separation filters (30 µm) on magnetic cell sorting separation columns (LS columns, Miltenyi Biotech, Bergisch Gladbach, Germany) binding magnetically labelled CD11b^+^ cells, which were subsequently isolated by removing the column from the magnetic device. The flow through of CD11b^−^ cells was additionally applied on a depletion column (LD columns, Miltenyi Biotech, Bergisch Gladbach, Germany) to remove remaining CD11b^+^ cells. Purity of CD11b^+^ and CD11b^−^ cells was analysed afterwards by flow cytometry using a CD11b-specific antibody (eBioscience). Purity ranged in the CD11b^+^ fraction from 81% to 99.5% and in the CD11b^−^ fraction from 93.7% to 98.9%, referring to total viable counts. The unselected and the CD11b^−^ depleted cell fractions were used for the GC sensitivity assay.

### Flow Cytometric analysis

5 × 10^5^ cells were incubated with fluorescently labelled antibodies (CD3, 17A2, Alexa Fluor 700, eBioscience; CD4, RM4-5, APC-eFluor 780, eBioscience; CD8a 53-6.7, Pacific Blue, BD Bioscience; CD19, 1D3, PE, BD Bioscience; CD11b, M1/70, Alexa Fluor 700, eBioscience) at 4 °C for 30 min. Following antibody incubation, cells were washed two times with FACS buffer (PBS, 10% FCS, 0.1% NaN3). Samples were analysed using LSRII flow cytometer (BD Biosciences, Heidelberg, Germany) and FlowJo software (Tree Star, Ashland, OR, USA). A total of 5 × 10^4^ events was analysed for each sample.

### Protein extraction

For analysis of GR expression, remaining splenocytes of the GC sensitivity assay were washed with phosphate buffered saline (PBS) and resuspended in an appropriate amount of cell extraction buffer (1 ml/10^8^ cells; Life Technologies GmbH, Darmstadt, Germany) supplemented with complete mini protease inhibitor (Roche Diagnostics GmbH, Mannheim, Germany), incubated for 30 min on ice and centrifuged for 15 min (1600 rpm, 4 °C). The supernatant was stored at −20 °C. Total protein concentrations were determined using a commercial kit (Bicinchoninic Acid Protein Assay Kit, Thermo Scientific, Rockford, USA).

### Western blotting

Western blotting was performed as described previously^28,43,45^ using equal amounts of protein lysates (20 µg) and antibody for rabbit anti-mouse GR (1:1500, Santa Cruz Biotechnology, Inc., Heidelberg, Germany) and goat anti-mouse FKBP51 (1:500, Santa Cruz Biotechnology, Inc., Heidelberg, Germany). After incubation with HRP-conjugated whole goat anti-rabbit (GR; 1:5000, Cell Signaling Technology, New England Biolabs GmbH, Frankfurt am Main, Germany) or donkey anti-goat (FKBP51, 1:6000, Santa Cruz Biotechnology, Inc., Heidelberg, Germany) secondary antibody, antibody binding was visualized on Molecular Imager® ChemiDoc™ MP system (Bio-Rad Laboratories, München, Germany) using a western blotting detection reagent (Bio-Rad Laboratories, München, Germany). Afterwards, each membrane was stripped using Re-Blot Plus Antibody Stripping Solution (Millipore GmbH, Schwalbach, Germany) and probed with primary rabbit anti-mouse glyceraldehyde 3-phosphate dehydrogenase (GAPDH) antibody (1:5000, Life Technologies GmbH, Darmstadt, Germany) as loading control. Visualization and digitization were performed as described above. Semiquantitative densitometric analyses of the signals were performed using Image Lab™ Software (Bio-Rad Laboratories, München, Germany). GR (~86 kDa) and FKBP51 (~51 kDa) protein expression for each mouse was normalized to GAPDH (~36 kDa) expression and averaged per group.

### Analysis of the severity of dermal and subdermal bite wounds

Following decapitation, the skin (with fur attached) of the CSC mice was removed so that skin and body were still connected at the tail root and the legs. Afterwards pictures depicting both the skin (dermal) and the body (subdermal) from all CSC mice were taken using identical camera position and settings. All pictures were then digitally overlayed with a standardized grid consisting of 20 squares (4 × 5 squares; each square 180 × 240 pixels; overall 720 × 1200 pixels) covering the skin and 20 squares (4 × 5 squares; each square 180 × 240 pixels; overall 720 × 1200 pixels) covering the body. Afterwards the severity of the received bite wounds was scored according to a developed score (see Supplementary Figure 1A), considering both size and intensity of wounds on the skin (dermal) and on the body (subdermal):

Body – affected area: 0, no bites; 1, <1/3 of the area; 2, 1/3–2/3 of the area; 3 > 2/3 of the area; maximum subscore: 60.

Body – intensity of injuries: 1, mild; 2, severe; maximum subscore: 40.

Skin – affected area: 0, no bites; 1, single bites; 2, several bites; 3 spacious bites; 4, bites in the whole square; maximum subscore: 80.

Skin – intensity of bite wounds: 1, mild; 2, moderate; 3, severe; 4, necrotic; maximum subscore: 180.

Skin – degree of purulence: 0, no purulence; 1, mild purulence; 2, severe purulence; maximum subscore: 40.

The total bite score of each mouse represents the sum of the skin (dermal) and the body (subdermal) and possible scores range from 0 to 300.

### Statistics

For statistical comparisons, the software package IBM SPSS statistics (version 22.0) was used. Kolmogorov-Smirnov test using Lilliefors’ correction was employed to test normal distribution of all acquired data sets. Outliers in normally distributed data sets were identified using Grubbs test and excluded from further analysis. Normally distributed data sets were subsequently analysed using parametric statistics, i.e. parametric Student’s *t*-test (one factor, two independent samples), two way ANOVA for repeated measures (two factors, two or more dependent samples). All tests comparing more than two samples were followed, when a significant main effect was found, by *post-hoc* analysis using Bonferroni pairwise comparison. Non-normally distributed data sets were analysed using non-parametric statistics, i.e. Mann-Whitney *U* test (one factor, two independent samples), Friedman ANOVA (one factor, more than two dependent samples). Normally distributed data are presented as bars (mean + SEM). Non-normally distributed data are presented as box plots. Solid line represents the median, dashed line represents the mean for each data set. Lower box indicates 25^th^, upper box indicates 75^th^ percentile. If n > 8 per group, 10^th^ (lower error bar), and 90^th^ percentile (upper error bar) as well as possible outliers beyond the percentiles (indicated by closed circles) are shown. The level of significance was set at *P* ≤ 0.05.

### Data availability

The data that support the findings of this study are available from the corresponding author on reasonable request.

## Electronic supplementary material

Supplementary Information
