# Supplementary material for: Splenic glucocorticoid resistance following psychosocial stress requires physical injury
Source: Sci Rep. 2017 Nov 16;7:15730. doi: 10.1038/s41598-017-15897-2 (PMC5691078; doi:10.1038/s41598-017-15897-2)
Supplement: Supplementary file 1 — Supplementary Information [file 41598_2017_15897_MOESM1_ESM.pdf]

Supplementary Information

Splenic glucocorticoid resistance following psychosocial stress requires physical injury

Sandra Foertsch[1#], Andrea M. Füchsl[2#], Sandra D. Faller[1], Hannah Hölzer[1], Dominik Langgartner[1], Joanna Messmann[3], Gudrun Strauß[3], Stefan O. Reber[1*]

[1]Laboratory for Molecular Psychosomatics, Clinic for Psychosomatic Medicine and Psychotherapy, University Ulm, Ulm, Germany

[2]Institute of Physiology, University of Regensburg, Regensburg, Germany

[3]Department of Pediatrics and Adolescent Medicine, University Medical Center Ulm, Ulm, Germany

[#] these authors contributed equally

* *Corresponding author and reprint requests:*

Prof. Dr. Stefan O. Reber, Laboratory for Molecular Psychosomatics, Clinic for Psychosomatic Medicine and Psychotherapy, University Ulm, Albert-Einstein-Allee 23, 89081 Ulm, Germany, Phone: 0049 731 50061943, Fax: 0049 731 50061927, mailto:stefan.reber@uniklinik-ulm.de

Supplementary Table 1: Behavioral patterns scored during CSC exposure.

Pro-Active Coping	Attacking	Defined as a jump at another individual with physical contact occurring.
	Mounting	Defined as standing with two forepaws on the back of another individual, frequently looking similar to a male-female copulation.
	Chasing	Defined as following a fleeing individual at running pace.
Re-Active Coping	Flight	Defined as directed movement away from another individual at running pace.
	Avoiding	Defined as directed movement away from another individual at walking speed, occurring before direct contact to the respective individual.
	Submissive upright posture	Defined as standing on the hind legs, raising the fore limbs and presenting the belly.
	Scouting	Defined as an attempt (stretching posture or exploring at walking speed) to figure out whether the danger (another individual) is still present; frequently seen when all subordinate mice are huddling together in a corner.
Received offensive Behavior	Attacks received	Defined as being jumped at by another individual with physical contact occurring.
	Mounts received	Defined as receiving the two forepaws of another individual on the back.
	Chasing received	Defined as being followed by another individual at running pace.

Shown are the definitions of single behavioral patterns scored in the present study to assess the individual coping strategy of each mouse during CSC exposure. These behavioral patterns have been chosen as they have been used in previous studies to assess rodent behavior during social encounters [1,2].

A

BODY		Size of affected area/square		Intensity of bite wounds/square
	0	No bites	1	Mild (rose)
	1	< 1/3 of the area	2	Severe (dark red, purulence)
	2	1/3 – 2/3 of the area		
	3	> 2/3 of the area		

SKIN		Size of affected area/square		Intensity of bite wounds/square		Degree of purulence
	0	No bites	1	Mild (rose)	0	No purulence
	1	Single bites	2	Moderate (red)	1	Mild purulence
	2	Several bites	3	Severe (dark red)	2	Severe purulence
	3	Spacious bites	4	Necrotic (black)		
	4	Bites in the whole square				

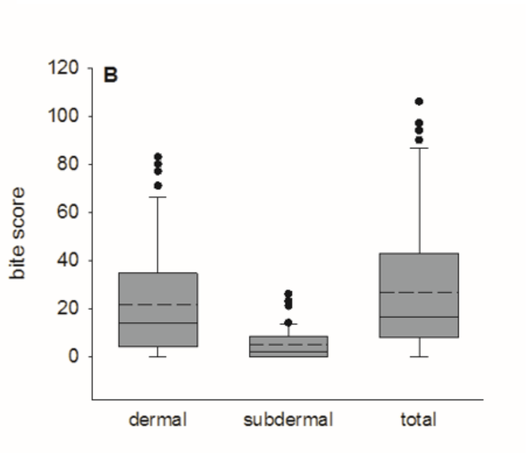

Supplementary Figure 1. Assessment of bite wounds in chronic subordinate colony (CSC) housed mice

Severity of dermal and subdermal bite wounds in CSC mice was assessed employing a bite score considering both size and intensity of dermal (skin, with fur attached) and subdermal (body) wounds according to the details shown in (A). The total bite score of each mouse represents the sum of the skin-score (ranging from 0 to 200) and the body-score (ranging from 0 to 100), with overall possible scores ranging from 0 to 300. The dermal, subdermal and total bite score of all CSC mice (n = 46) is depicted in (B). ■ CSC.

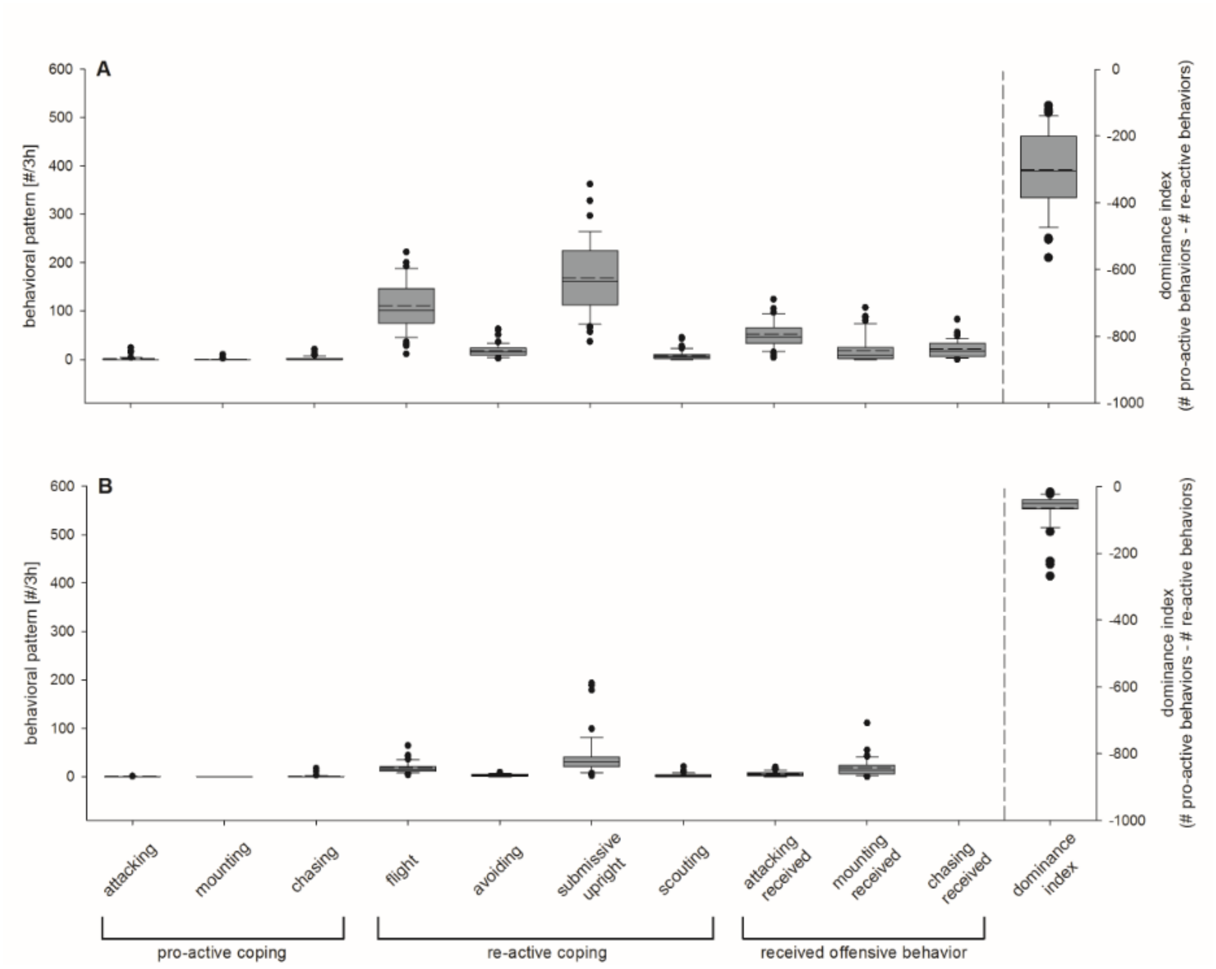

Supplementary Figure 2. Assessment of individual behavioral patterns in the morning and evening during chronic subordinate colony housing (CSC)

Pro-active, re-active and received offensive behavior shown during CSC exposure was assessed from 10 to 11 am in the morning, as well as from 5 to 6 pm in the evening on days 1, 8 and 15 of CSC exposure, allowing calculation of a Dominance Index (DI) for each CSC mouse. Each behavioral pattern was analysed in CSC mice (n = 46) separately in the morning (A) and the evening (B). ■ CSC.

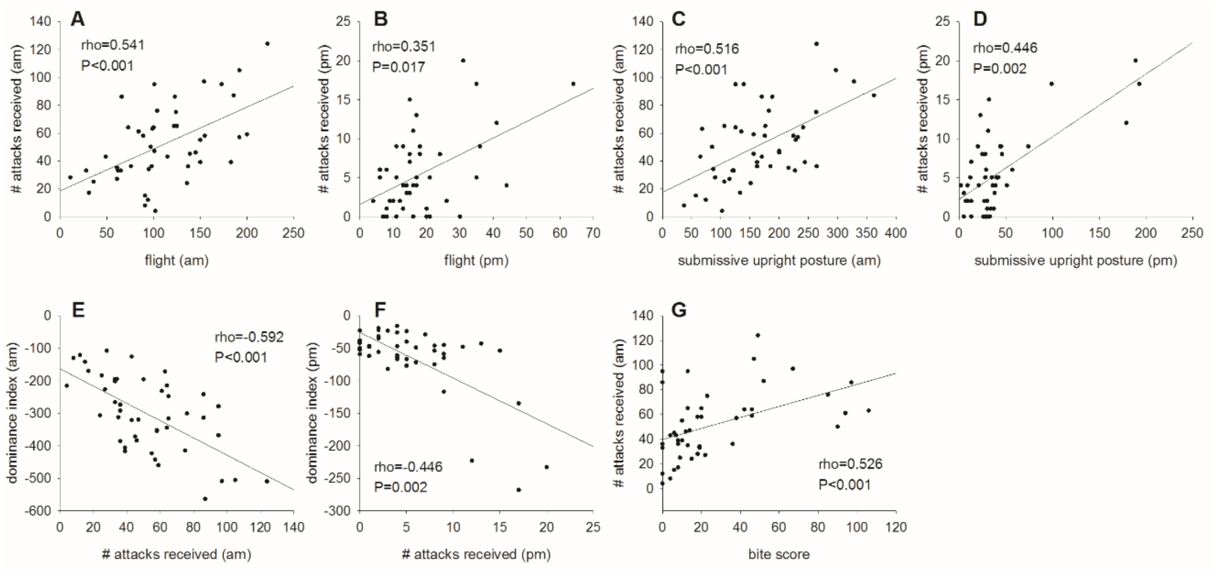

Supplementary Figure 3. Correlation analyses – Part III

Depicted are significant correlations within individual behavioral coping patterns shown in the morning (am) and/ or in the evening (pm) on days 1, 8 and 15 and the bite scores in all CSC (n = 46) mice. The number of attacks received was significantly positive correlated with, flight in both, the morning (A) and the evening (B), as well as with submissive upright posture in the morning (C) and the evening (D). A significant negative correlation was shown between the dominance index (DI) and the number of attacks received in the morning (E), as well as in the evening (F). Furthermore, the bite score positively correlated with the number of attacks received in the morning (G).

Full Western blots for Figure 3K and 3L

Figure 3K

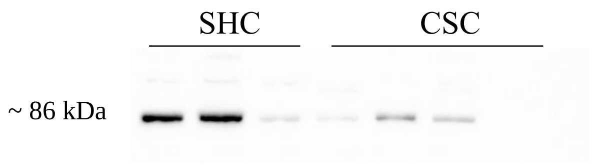

GR Gel 1

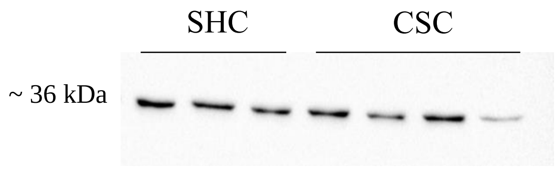

GAPDH Gel 1

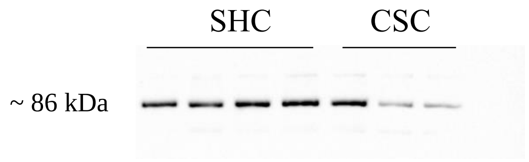

GR Gel 2

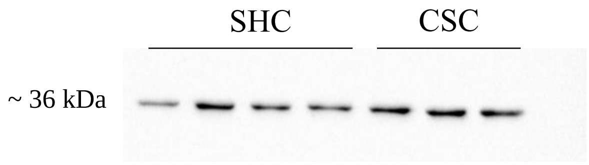

GAPDH Gel 2

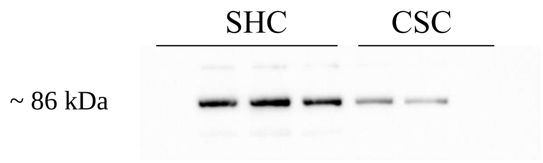

GR Gel 3

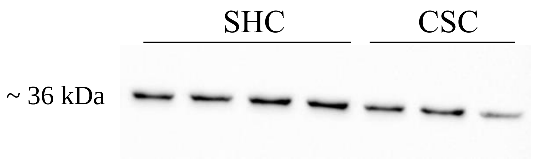

GAPDH Gel 3

Single-housed control (SHC) mice; chronic subordinate colony (CSC) housed mice.

Figure 3L

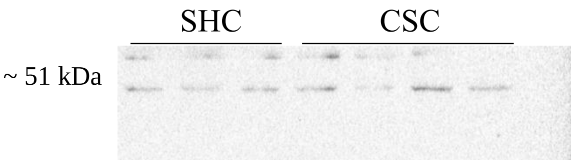

FKBP51 Gel 1

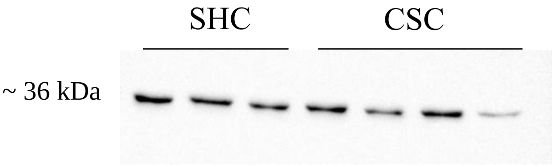

GAPDH Gel 1

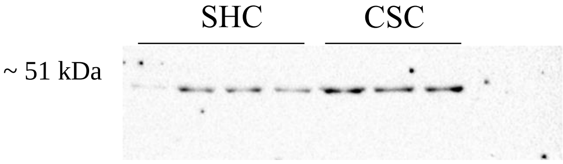

FKBP51 Gel 2

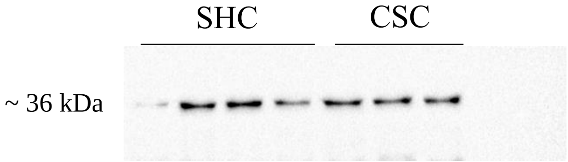

GAPDH Gel 2

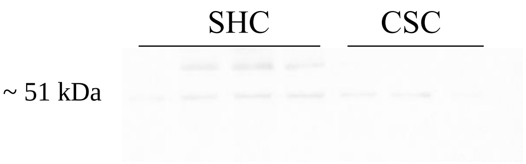

FKBP51 Gel 3

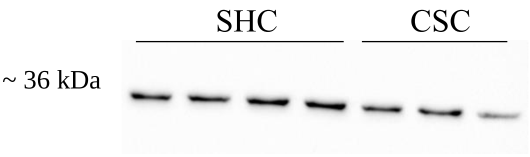

GAPDH Gel 3

Single-housed control (SHC) mice; chronic subordinate colony (CSC) housed mice.

References

1 Stefanski, V., Peschel, A. & Reber, S. Social stress affects migration of blood T cells into lymphoid organs. *Journal of neuroimmunology* **138**, 17-24 (2003).

2 Reber, S. O. & Neumann, I. D. Defensive behavioral strategies and enhanced state anxiety during chronic subordinate colony housing are accompanied by reduced hypothalamic vasopressin, but not oxytocin, expression. *Annals of the New York Academy of Sciences* **1148**, 184-195 (2008).
